# Hydrogen Generation from the Hydrolysis of Diamond-Wire Sawing Silicon Waste Powder Vibration-Ground with KCl

**DOI:** 10.3390/molecules30020223

**Published:** 2025-01-08

**Authors:** Zhicheng Li, Tao Zhou, Jiangfan Liao, Xiufeng Li, Wenhui Ma, Guoqiang Lv, Shimin Zhao

**Affiliations:** 1 Faculty of Metallurgical and Energy Engineering, Kunming University of Science and Technology, Kunming 650093, China; lzc18508890175@163.com (Z.L.); zt18765982961@163.com (T.Z.); ljf18119079502@163.com (J.L.); kglxf@kust.edu.cn (X.L.); mawenhui@ynu.edu.cn (W.M.); 2 School of Engineering, Yunnan University, Kunming 650500, China; 3 Yunnan Key Laboratory for Pollution Processes and Control of Plateau Lake-Watersheds, Yunnan Institute of Ecological and Environmental Sciences, Kunming 650034, China

**Keywords:** photovoltaic silicon waste, hydrolysis, hydrogen production, kinetics

## Abstract

Diamond-wire sawing silicon waste (DSSW) derived from the silicon wafer sawing process may lead to resource waste and environmental issues if not properly utilized. This paper propounds a simple technique aimed at enhancing the efficiency of hydrogen production from DSSW. The hydrolysis reaction is found to become faster when DSSW is ground. Among the studied grinding agents, KCl has the best performance. The grinding duration and addition amount remarkably affect the final hydrogen yield and initial hydrogen generation rate (IHGR). Among all studied samples, DSSW-KCl 25 wt% ground for 3 min shows the best performance with a hydrogen yield of 86.1% and an IHGR of 399.37 mL min^−1^ (g DSSW)^−1^ within 650 s. The initial temperature is also found to have a significant influence on the hydrolysis of the DSSW-KCl mixture, and the reaction can proceed to 85% conversion in 100 s with an IHGR of 1383.6 mL min^−1^ (g DSSW)^−1^ at 338 K. The apparent activation energy for the hydrolysis reaction of the DSSW-KCl composite powder was found to be 45.62 kJ mol^−1^ by means of an Arrhenius plot. The rate-determining step for the rapid reaction of DSSW to produce hydrogen is chemical reaction control, while the slow reaction is controlled by diffusion.

## 1. Introduction

With the ongoing advancement of industrialization, the demand for energy is escalating, resulting in a range of environmental and climate challenges; thus, there is an urgent need for renewable clean energy as a viable alternative in the future [1,2]. Hydrogen is unequivocally a novel clean energy carrier that offers advantages in terms of storage and transportation [3], and it can be directly utilized in portable energy systems as well as hydrogen fuel cells [4]. In nature, hydrogen predominantly exists in compound form within water, while traditional methods of hydrogen production primarily involve fossil fuels, industrial by-products, and the electrolysis of water [5]. However, the hydrogen generated through these processes must be stored and transported prior to delivery to end users, a procedure that entails certain inherent risks due to the highly flammable and explosive nature of hydrogen gas. Consequently, the rapid production and safe storage and transportation of hydrogen have emerged as significant barriers to the advancement of hydrogen energy [1]. In order to address these challenges, researchers have introduced a range of innovative methods for hydrogen production. Among these approaches, the hydrolysis of reactive metals, alloys, and compounds for hydrogen production has emerged as a prominent area of research [6,7], particularly concerning aluminum and its alloys. All of these reactions necessitate the involvement of water, which serves as a vital resource abundant in hydrogen [8]. However, owing to its abundance in the Earth’s crust and other advantages, including a high hydrogen capacity, non-metallic silicon is regarded as a more promising material for hydrogen production compared to aluminum [9]; consequently, silicon is viewed as a more attractive option for hydrogen storage and conversion. Furthermore, the byproduct of silicon in the hydrolysis reaction is silica, which can be recovered via a carbon reduction process.

Silicon has the potential to generate hydrogen gas through hydrolysis; however, its surface is prone to forming a dense oxide layer that hinders further interaction between water and silicon, resulting in negligible reactivity in neutral aqueous solutions [10]. To mitigate the effects of the passive oxide layer and improve the reactivity of the hydrolysis reaction, researchers have implemented strategies such as utilizing alkaline solutions, reducing particle size, fabricating alloys, and developing silicon-based composites. However, the reactions occurring in alkaline solutions exhibit significant corrosiveness, rendering this method impractical for onboard hydrogen production. Yoo et al. [11] investigated the production of Al-Si alloy powder via centrifugal atomization with varying silicon content, revealing that the Al-Si alloy can rapidly hydrolyze to generate hydrogen at room temperature in a 30 wt% NaOH solution. The malleability of aluminum leads to the encapsulation of silicon, thereby inhibiting its participation in the reaction and resulting in a substantial amount of unreacted silicon remaining in the solution. Erogbogbo et al. [12] investigated the synthesis of silicon nano-powders with reduced sizes (approximately 10 nm) through CO_2_ laser thermal decomposition within custom-designed stainless steel prototype containers containing silicon nano-powders, achieving hydrogen gas production that exceeds 2 mol H_2_ per mole of theoretical yield. However, this preparation method is characterized by complexity and high costs, which hinder its industrial applicability. Brack et al. [13] examined the influence of varying volumes of NaOH solution on the hydrogen production performance during the hydrolysis of silicon powder. The results indicated that an increase in NaOH volume led to a reduction in the induction period, which was attributed to the enhanced thermal effects of the reaction. However, as the volume continued to rise, changes in hydrogen production began to stabilize, making it challenging to achieve the widely reported 14 wt% weight hydrogen storage efficiency. Furthermore, a range of novel composite materials has been investigated, including silicate systems and silane-hydride systems. Researchers employed mechanical ball milling and compaction techniques to activate silicon, facilitating the formation of various surface defects that mitigate the effects of a dense oxide layer. Kalamkar et al. [14] conducted an in-depth study on the ball milling process of LiH, NaH, CaH_2_, and TiH_2_ with silicon additives, demonstrating that the activation energy decreased as well and the hydrogen desorption temperatures were, respectively, reduced to 523 K, 453 K, 488 K, and 463 K. Concurrently, Dann et al. [15] employed a compacting and pelletizing method to eliminate the surface oxide layer, thereby reducing the induction period, and observed that the hydrogen generation rate of the samples was significantly enhanced following the addition of sodium chloride and sodium polyacrylate. While the aforementioned methods effectively enhance the reactivity of silicon hydrolysis, they predominantly utilize industrial-grade silicon powder. Utilizing silicon-containing industrial by-products as raw materials could facilitate a “waste-to-treasure” approach. With the rapid advancement of silicon-based solar cell technology, the demand for silicon wafers has been steadily increasing. The diamond wire sawing process for silicon wafers results in the generation of 28% DSSW, which may lead to resource waste and environmental issues if not properly utilized. Consequently, the utilization of DSSW hydrolysis for hydrogen production can mitigate environmental pollution and the waste of silicon resources and reduce the cost of hydrogen production.

Existing research indicates that silicon powder can be significantly activated through mechanical activation via ball milling [16,17,18] or by reaction in alkaline solutions [19,20]. Nevertheless, there is currently a lack of reports regarding the hydrolysis reaction of DSSW-KCl composite powder under alkaline conditions. Drawing inspiration from the research conducted by Wang et al. [21] and Dann et al. [15], this study seeks to utilize the DSSW-KCl composite powder for water hydrolysis aimed at hydrogen production. To reduce the particle size of DSSW powder, increase the reaction contact area, and enhance hydrolysis reaction activity, this study employed a vibration grinding method to prepare DSSW-X (grinding agent) composite powder. Furthermore, it investigated the effects of grinding agent type, addition amount, and vibration grinding duration on the hydrolysis reaction kinetics of DSSW-KCl composite powder.

## 2. Results and Discussion

The hydrolysis reaction of DSSW in the presence of sodium hydroxide can be represented as follows:

(1) $$\mathrm{Si}+2{\mathrm{NaOH}+H}_{2}O\to {\mathrm{Na}}_{2}{\mathrm{SiO}}_{3}+2H_{2}$$

Based on the aforementioned reaction, the effects of grinding agent type, grinding duration, KCl content, and initial temperature on the hydrolysis reaction of DSSW were investigated.

### 2.1. Effect of Grinding and Abrasives

**Figure 1:**
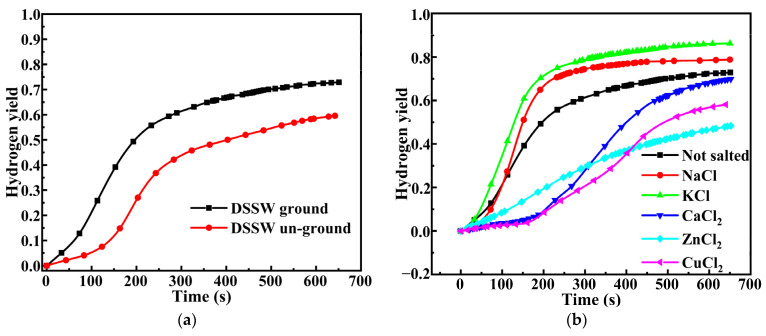
(**a**) DSSW vibration grinding for 180 s and un-ground hydrogen production curves and (**b**) hydrogen production curves of different abrasive samples ground for 180 s.

To assess the effect of vibration-ground on the hydrolysis reaction, we compared ground and un-ground samples. Figure 1a shows the hydrogen generation curves of un-ground DSSW and DSSW ground for 180 s. The DSSW hydrolysis reaction is found to become faster and more intense when DSSW is ground. The ground and un-ground samples show a hydrogen yield of 72.89% and 59.28% with an IHGR of about 238 and 72 mL min^−1^ (g DSSW)^−1^ at 650 s. Vibratory grinding has a remarkable promoting influence on the hydrogen yield and the IHGR from the hydrolysis of DSSW.

**Figure 2:**
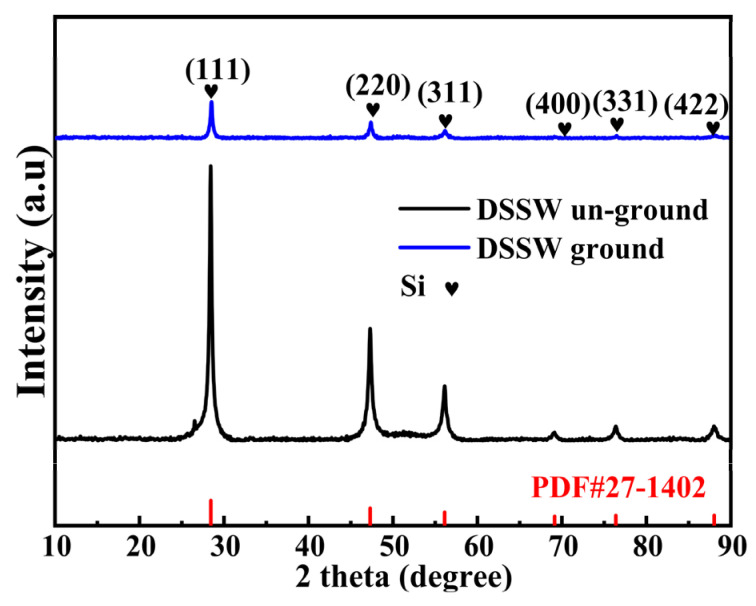
XRD patterns of DSSW vibration-ground for 180 s and un-ground.

**Figure 3:**
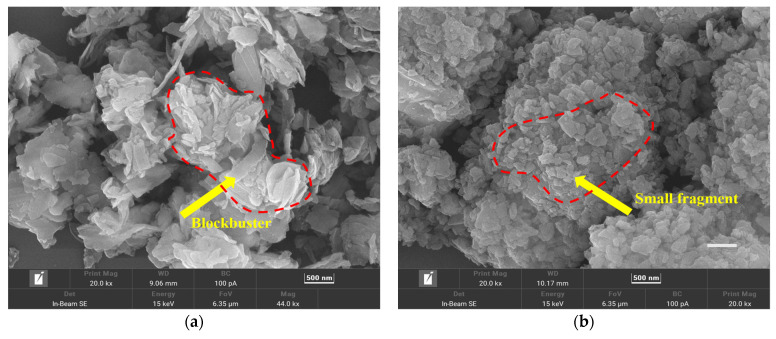
SEM micrograph of (**a**) un-ground DSSW powder, (**b**) DSSW powder vibration-ground for 180 s.

Figure 2 shows the XRD patterns of DSSW powder un-ground and ground for 180 s, respectively. The diffraction characteristic peaks of Si are obvious, and no diffraction peaks of other substances were detected. Broadened and weakened peaks are also noticed in the patterns because of the decrease in Si crystallite size and the accumulation of microstrains. An intriguing change in the relative intensity of the (111), (220), and (311) peaks is observed in Figure 3. Notably, the (400), (331), and (422) crystal planes show almost no corresponding diffraction peaks. The DSSW particles underwent a transition from large blockbuster structures (Figure 3a) to smaller fragments (Figure 3b), resulting in a reduction in particle size and an increase in the contact area of the DSSW powder, thereby enhancing the activity of the hydrolysis process. However, in Figure 3b, particle agglomeration is observed. Researchers have proposed the incorporation of brittle salts during the grinding process in order to alleviate agglomeration and enhance activity [22]. This study aims to employ this approach to achieve similar effects. Consequently, 25 wt% (KCl, CaCl_2_, NaCl, ZnCl_2_, and CuCl_2_) were added to DSSW powder, the mixture was ground for 180 s, and the hydrogen generation profiles of the samples were obtained and compared.

Figure 1b shows the generation profiles of DSSW vibration-ground with different grinding agents for 180 s. The sample with KCl shows the highest yield of 86.1% with an IHGR of about 399.37 mL min^−1^ (g DSSW)^−1^. The IHGRs of samples added with NaCl, CaCl_2_, ZnCl_2_, and CuCl_2_ are 332.33, 55.67, 112.67, and 42.67 mL min^−^^1^ (g DSSW)^−^^1^, respectively. The sample with KCl clearly shows the best performance (highest hydrogen yield and IHGR) among the tested grinding agents. The advantages of KCl over other grinding agents can be delineated as follows: Firstly, when the DSSW-KCl composite powder is dissolved in NaOH solution, it generates a mixed alkali solution (KOH and NaOH), wherein KOH exhibits greater basicity than NaOH, thereby exerting a more pronounced promoting effect on the reaction. Furthermore, during the DSSW hydrolysis reaction conducted at elevated alkali concentrations, OH^−^ serves as the reactant [23]. It is essential to note that under these circumstances, both KCl and NaCl can be completely dissolved without precipitation; however, the cations from CaCl_2_, ZnCl_2_, and CuCl_2_ react with hydroxide ions to form precipitates, which subsequently reduces the concentration of free hydroxide ions and impedes the further advancement of the reaction. Although these grinding agents possess a certain degree of dissolution heat when dissolved in water, the principal reaction of the DSSW-X composite powder is with an alkali solution [23]. Secondly, the chloride ions present in the grinding agent can induce pitting corrosion on silicon particles [24], thereby enhancing the surface area of fresh silicon particles. Consequently, during the hydrolysis of silicon waste, potassium hydroxide formed from the interaction between potassium ions and hydroxide ions in solution, along with chloride ions that contribute to a synergistic effect, positions KCl as the most effective grinding agent in this study. Notably, Dann et al. [15] investigated the impact of NaCl addition on hydrolysis-based hydrogen production, where a Si-NaCl 50 wt% sample yielded less than 55% hydrogen within 600 s, whereas the DSSW-NaCl 25 wt% sample in this study achieved a hydrogen yield of 78%. This disparity might be ascribed to the differences in process conditions (Dann employed powder compaction for pellet production, whereas this study utilized vibrating grinding).

### 2.2. Effect of Grinding Duration

**Figure 4:**
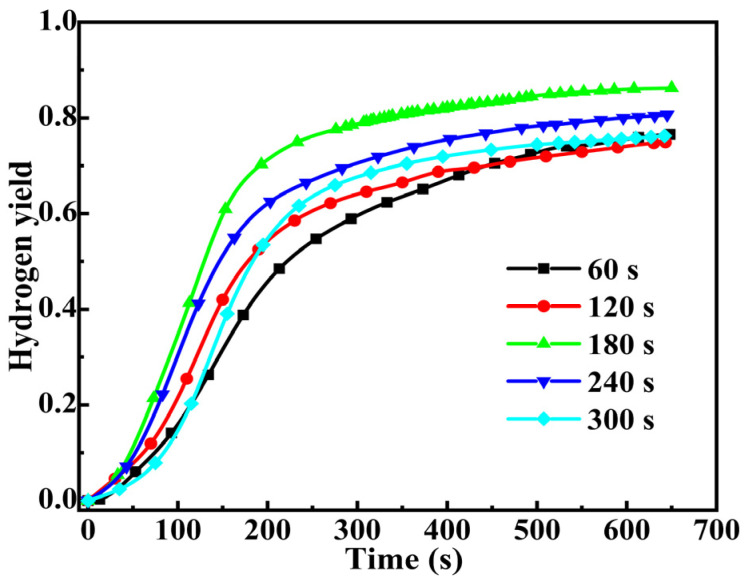
Hydrogen production curves for DSSW-KCl 25 wt% powder ground for various durations.

The hydrolysis of DSSW-KCl at a concentration of 25 wt% under varying grinding durations is illustrated in Figure 4. Figure 4 indicates that a brief induction period exists at the onset and all curves similarly display rapid H_2_ release at the initial reaction time. As the reaction progresses, the concentration of the base decreases, and the reaction rate decreases accordingly. The grinding time significantly influences the reaction characteristics of the DSSW-KCl samples. With increased grinding time, the hydrogen yield initially increases and then decreases. The sample ground for 180 s has the highest yield of 86.1% within 650 s and IHGR of 399.37 mL min^−1^ (g DSSW)^−1^. In comparison, the IHGR values for samples ground for 60, 120, 240, and 300 s are recorded at 154.46, 232.01, 364.64, and 142.92 mL min^−1^ (g DSSW)^−1^.

**Figure 5:**
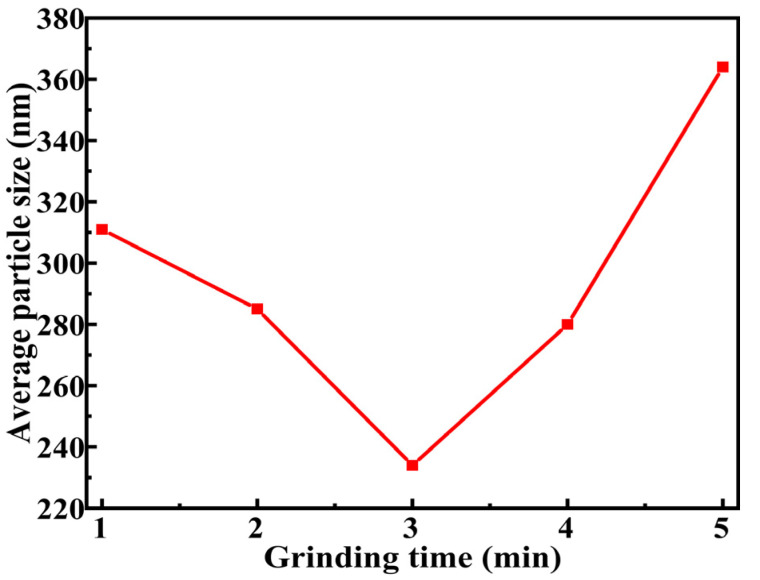
Images of average particle size for DSSW-25% wt KCl samples ground for varying durations.

Figure 5 shows that the particle size varies with grinding duration. Specifically, the grinding time significantly influences particle size. With increased grinding time, the particle size initially decreases and then increases, achieving its minimum particle size after 180 s of grinding. This reduction in particle size results in an increased specific surface area, thereby enhancing the hydrolysis reaction activity of DSSW and yielding optimal hydrogen production performance under these conditions. However, extending the grinding time under non-vacuum conditions led to a reduction in hydrogen production rate, which can be attributed to the cold-welding effect that induces partial oxidation and agglomeration [21]. This phenomenon results in an increase in particle size, subsequently causing a decline in hydrogen production performance for the samples ground for 240 and 300 s.

**Figure 6:**
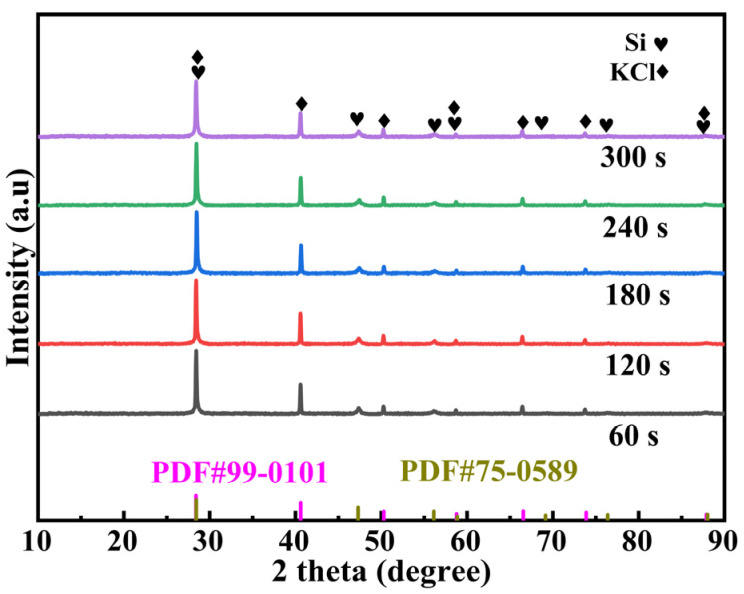
X-ray diffraction patterns of DSSW-KCl 25 wt% powder ground for various durations.

**Figure 7:**
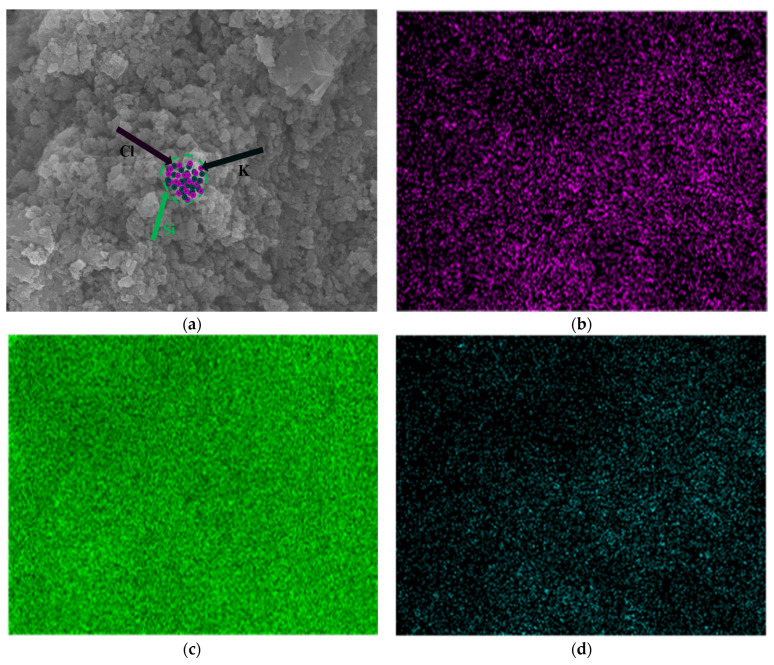
(**a**) Micrograph of DSSW-KCl 25 wt% vibration-ground for 180 s; mapping diagram of DSSW-KCl 25 wt% vibration-ground for 180 s. (**b**) K element, (**c**) Si element, (**d**) Cl element.

Figure 6 shows that the diffraction characteristic peaks of Si and KCl are obvious, and no additional phase diffraction peaks were detected. During the grinding process, KCl was incorporated into the crystal lattice of DSSW, resulting in overlapping diffraction peaks for Si(111), (222), and (422) and KCl(200), (400), and (440). It is notable that the influence of grinding time on the intensity of these diffraction peaks was found to be insignificant. Although KCl diffraction peaks were identified in the XRD pattern, the overlapping of Si and KCl diffraction peaks necessitated an investigation into the distribution of KCl within DSSW. To elucidate the reasons behind the optimal hydrogen production observed in the sample ground for 180 s, SEM-EDS mapping analysis was carried out on the DSSW 25 wt% KCl sample ground for 180 s, and the results are shown in Figure 7. The images show that KCl particles are homogeneously distributed around DSSW powder and may be embedded into it during grinding. The sample exhibited optimal hydrolysis performance, which can be summarized as follows: (1) the brittleness of KCl resulted in the formation of numerous cracks and rough surfaces during grinding, thereby increasing the specific surface area of the particles; (2) KCl formed a protective layer on the surface of the DSSW, effectively reducing oxidation in air [25] and preserving the material’s reactivity; (3) the KCl embedded on the Si particle surfaces dissolved in NaOH solution, creating multiple reaction channels that enhanced hydrolysis reaction activity [8].

### 2.3. Effect of KCl Content

**Figure 8:**
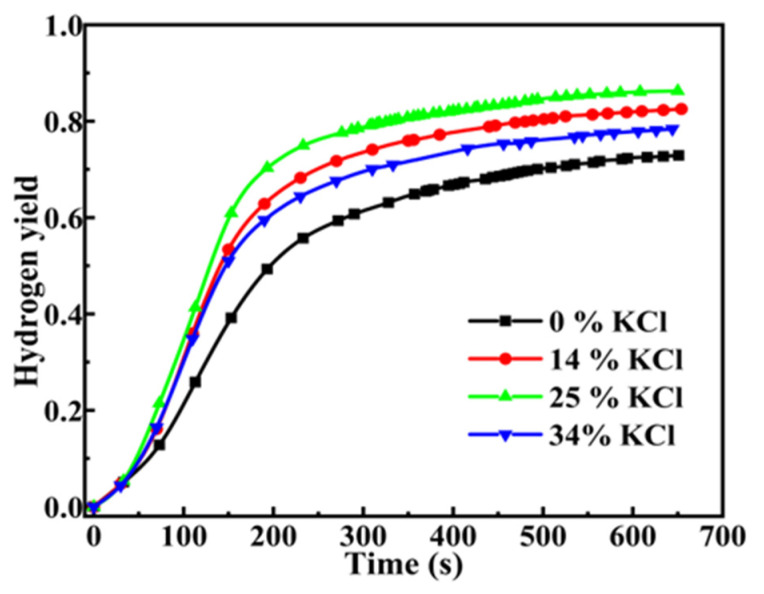
Hydrogen production curves for DSSW powder added to different amounts of KCl and vibration-ground for 180 s.

The hydrolysis of DSSW-KCl at a grinding duration of 180 s under varying additions of KCl is illustrated in Figure 8. Figure 8 indicates that the addition of KCl has a considerable influence on the performance of the hydrolysis reaction, and the trend of image changes is comparable to that presented in Figure 5. With increased KCl content, the hydrogen production performance initially increases and then decreases. At a KCl addition level of 25% wt, the hydrogen yield reached its highest value of 86.1% within 650 s, the IHGRs of these samples are 238.52, 313.07, 399.37, and 319.78 mL min^−1^ (g DSSW)^−1^ for KCl additions of 0%, 14%, 25%, and 34% wt.

**Figure 9:**
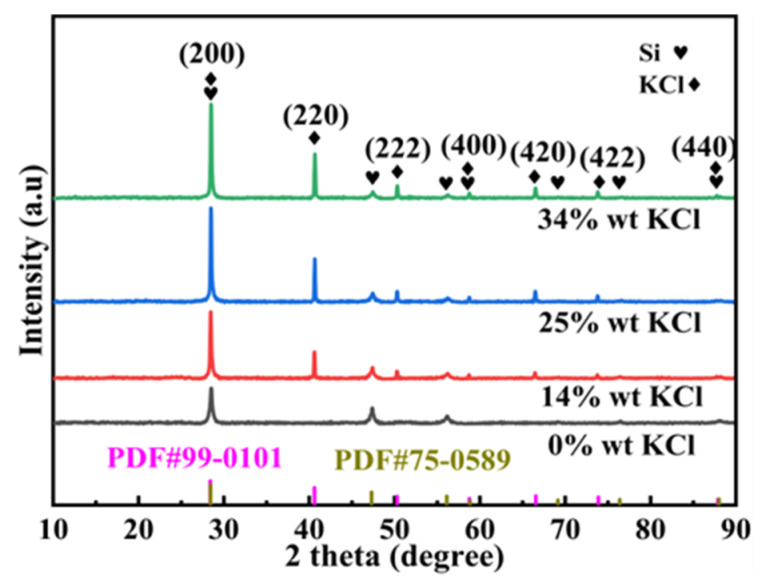
X-ray diffraction patterns of DSSW-X wt% KCl (x = 0, 14, 25, 34) vibration-ground for 180 s.

**Figure 10:**
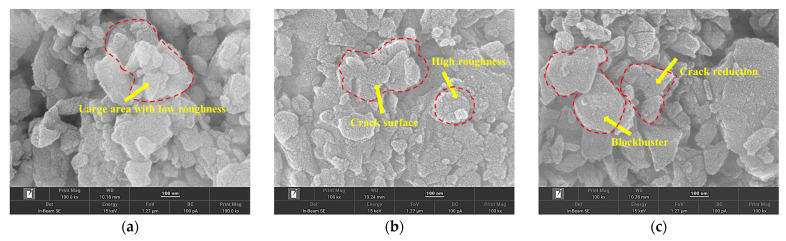
SEM images of DSSW-KCl samples subjected to vibration grinding for 180 s with varying KCl concentrations of (**a**) 0% wt, (**b**) 25% wt, and (**c**) 34% wt.

Figure 9 shows that the diffraction peaks of Si and KCl are distinctly observable, while no diffraction peaks from other substances are detected. The embedding of the crystal lattices of Si(111), (222), and (422) and KCl(200), (400), and (440) gives rise to the phenomenon of overlapping diffraction peaks. Notably, the intensity of the diffraction peaks for Si(111) and KCl(200), (220) increases with rising KCl content; when the KCl content reaches 25%, the diffraction peak intensity tends to stabilize. The observation outcomes indicate that when the KCl content attains 25 wt%, the intercalation between DSSW and KCl has reached a critical state, and any further increase might have adverse effects (Figure 10c). Additionally, with increased KCl content, the intensity of the Si(220) diffraction peak reduces, indicating a preferred orientation along the c-axis [22]. This orientation gives rise to the crystal being more prone to deformation, with the energy requisite for deformation along the c-axis being conspicuously lower than in other directions. This engenders a weakening of the (220) peak intensity and an augmentation in the intensity of the (111) peak.

Figure 10 shows that increased KCl content leads to a reduction in the size of DSSW particles and an enhancement in surface roughness. This phenomenon can be attributed to the brittleness of KCl, which acts as a cutting tool for silicon particles during grinding, and the cutting effect becomes more pronounced with increased KCl content. Furthermore, the encapsulation of silicon particles by KCl can diminish the occurrence of cold-welding during grinding [21], reduce the size of DSSW, and enhance the surface roughness of the particles. However, when the addition of KCl rose to 34 wt%, the roughness of the surface declined and the particle size was augmented. The elevated KCl concentration resulted in a more pronounced cutting action on the DSSW, leading to a reduction in silicon particle size and an increase in surface energy, which enhanced the interactions between substances. Consequently, this further promoted the embedding of KCl within the DSSW matrix, thereby bringing about a reduction in the KCl content on the surface of DSSW particles. This phenomenon further intensifies the cold-welding effect during the grinding process, exacerbates the crystal agglomeration, and gives rise to an increase in particle size and a decrease in surface roughness. The specific surface areas of the samples with 25 wt% and 34 wt% are 19.613 and 13.172 m^2^ g^−1^, respectively. This further elucidates why the hydrogen production performance of DSSW-KCl at 34 wt% is inferior to that of DSSW-KCl at 25 wt%; the latter possesses a more pronounced surface roughness and cracks, which enhance the sample’s activity and render it the optimal performer under these circumstances. Indeed, it has exceeded the activity reported by Kobayashi et al. [23] when nanoscale DSSW was employed for hydrolysis reactions and the hydrogen production per gram of DSSW has exceeded 400 mL.

### 2.4. Effect of Initial Temperature

**Figure 11:**
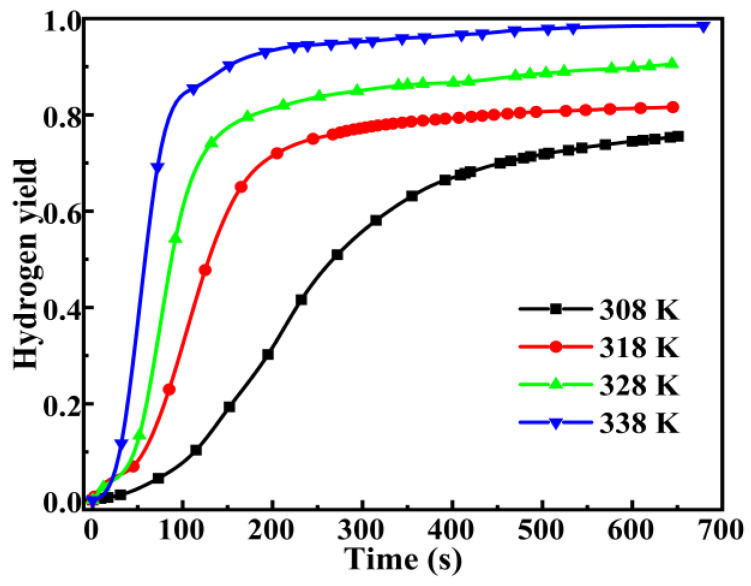
Hydrogen production curves of DSSW-KCl 25% wt samples subjected to vibration grinding for 180s at various temperatures.

The temperature exerts a remarkable influence on the kinetics of hydrolysis reactions, and the hydrolysis of magnesium, aluminum, and silicon [23,26,27,28] has been extensively studied. However, the interplay between the exothermic effect of DSSW hydrolysis and the initial temperature renders the apparent activation energy and the control step for hydrogen production through DSSW-KCl composite powder hydrolysis indistinct [15]. Furthermore, experiments were performed on the DSSW-KCl 25 wt% sample ground for 180 s at initial temperatures of 308, 318, 328, and 338 K, as shown in Figure 11.

**Figure 12:**
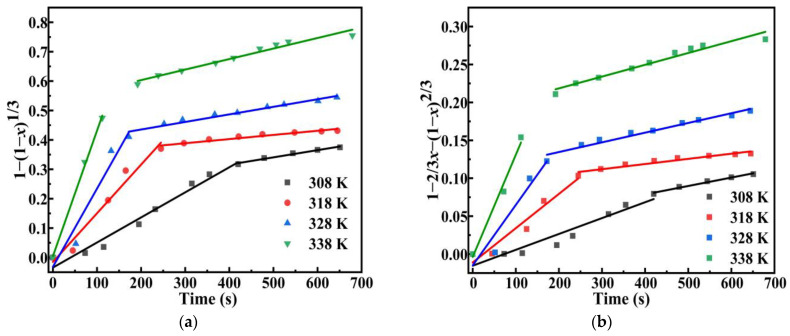
Fitted representations of the DSSW-KCl 25% wt samples at various temperatures based on the (**a**) chemical control model and (**b**) diffusion control model.

Figure 12 shows that the sample at initial temperatures of 308, 318, 328, and 338 K of hydrogen yield reached values of 75%, 81%, 90%, and 98% within 650 s. The IHGRs of these samples are 81.55, 399.37, 825.99, and 1383.6 mL min^−1^ (g DSSW)^−1^. The results conformed to our expectations, with both hydrogen yield and IHGR manifesting an upward trend as the initial temperature rose. However, at an initial temperature of 338 K, 85% of the hydrogen yield was attained within merely 100 s. The hydrolysis process for hydrogen production can be categorized into two distinct stages: the first stage represents the initial phase of a rapid reaction, while the second stage is characterized by a gradual deceleration as the alkali concentration diminishes. The hydrogen generation reaction between DSSW-KCl composite powder and NaOH solution is a typical solid–liquid heterogeneous reaction. For most solid–liquid heterogeneous reaction processes, the classic shrinking core non-reactive core model can be adopted [29]. The chemical reaction rate in this model is generally divided into two rate-determining steps: interfacial chemical reaction control and diffusion control. The kinetic equation can be written as follows [30]:

(2) $$1-{\left(1-x\right)}^{\frac{1}{3}}=k_{1}t$$

(3) $$1-\frac{2}{3}x-{\left(1-x\right)}^{\frac{2}{3}}=k_{2}t$$

*x* is the hydrogen yield, t is the reaction time, and *k*_1_ and *k*_2_ are the apparent reaction rate constants.

To derive the kinetic parameters and control steps for the hydrolysis of DSSW in hydrogen production, the experimental data of the hydrolysis of DSSW for hydrogen production in Figure 11 were substituted into Formulas (2) and (3) for fitting, and the results are shown in Figure 12. The apparent reaction efficiency constant (k) and correlation coefficient (R^2^) are shown in Table 1. Analysis of Figure 12 and the fitting data in Table 1 reveals that the fitting coefficients for the rapid reaction stage exceed 0.925, indicating adherence to a chemical reaction control model, where interfacial chemical reaction rates serve as the rate-determining step. Conversely, fitting coefficients for the slow reaction stage are all above 0.91, suggesting compliance with a diffusion control model, wherein the diffusion rate of OH^−^ to the surface of Si particles becomes the rate-determining factor.

The apparent activation energy of the hydrolysis of the DSSW-KCl composite powder process can be obtained according to Arrhenius law:

(4) $$k=A\exp\left(\frac{E_{a}}{\mathrm{RT}}\right)$$

**Figure 13:**
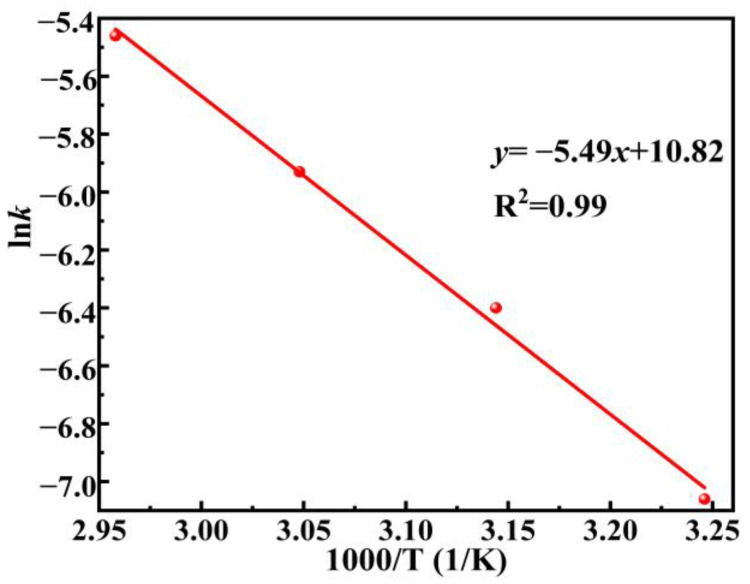
ln*k* and 1000/T Arrhenius fitting line.

In Formula (4), *k* is the apparent reaction rate constants at temperature T, *A* is the pre-exponential factor, and *E_a_* is the activation energy required for the chemical reaction control. According to the Arrhenius formula, take the logarithm of the apparent chemical reaction rate constant ln*k* and 1000/T to make a straight line, as shown in Figure 13. The activation energy estimated from the slope in Figure 13 was 45.62 KJ mol^−1^, and the pre-exponential factor A was 5 × 10^4^. Consequently, the macroscopic kinetic equation for the hydrogen production from the DSSW-KCl composite powder hydrolysis is as follows:

$$1-{\left(1-x\right)}^{\frac{1}{3}}=5\times 10^{4}\times \exp\left(\frac{45.62}{\mathrm{RT}}\right)\times t$$

## 3. Materials and Methods

### 3.1. Sample Preparation

**Figure 14:**
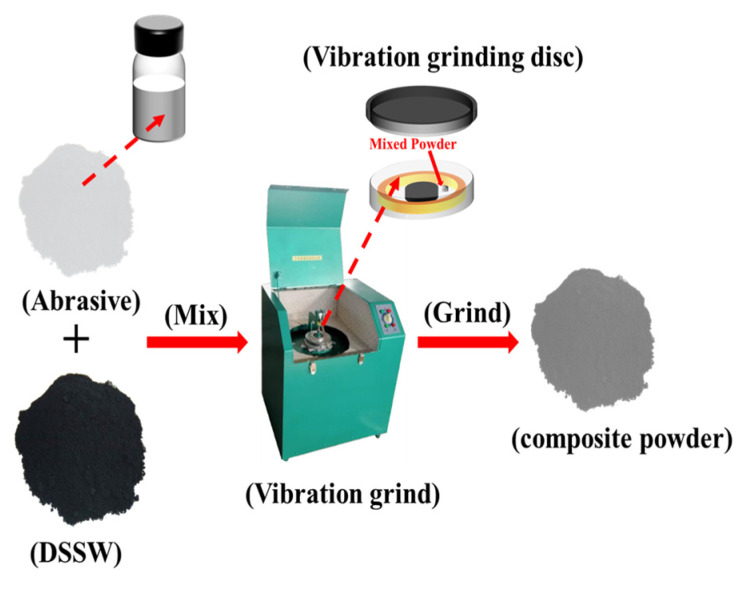
Illustrates the schematic representation of the preparation process for composite powders.

DSSW (single crystal silicon enterprise, purity 92.2%), NaOH (AR, original Tianjin Chemical Reagents Third Factory, Tianjin, China), deionized water (prepared by ourselves), KCl (AR, Chongqing Chuandong Chemical (Group) Co., Ltd., Chongqing, China), NaCl (AR, original Tianjin Chemical Reagents Third Factory), CaCl_2_ (AR, Sichuan Xilong Science Co., Ltd., Chengdu, China), ZnCl_2_ (AR, Shanghai McLin Bio-Tech Co., Ltd., Shanghai, China), and CuCl_2_ (AR, original Tianjin Chemical Reagents Third Factory) were obtained. In a vibrating grinding machine (model: GJ-1B, Lianqing City Tiananguan Instrument and Meter Co., Ltd., Tianjin, China), DSSW and the grinding agent were mixed and ground. The process is shown in Figure 14. The ground sample was transferred to a vacuum-sealed bag and stored in a vacuum-drying oven until use. Finally, the obtained sample was subjected to a hydrogen production experiment.

### 3.2. Sample Characterization

X-ray diffraction (XRD) measurements of DSSW and DSSW-KCl under various conditions were performed using a Rigaku Smart-Lab SE diffractometer from Rigaku, Tokyo, Japan. Scanning electron microscopy (SEM) images were acquired with a TESCAN MIRA LMS from the TESCAN, Brno, Czech Republic. The distribution of KCl within the ground and mixed powders was analyzed through energy-dispersive X-ray spectroscopy (EDS). Additionally, particle size was determined using a nanoparticle size analyzer.

### 3.3. Hydrolysis Experiment

Unless otherwise stated, the hydrolysis reaction was conducted at 318 K and atmospheric pressure using a self-assembled hydrolysis hydrogen generation system. Approximately 0.03 g of DSSW was combined with 10 mL (0.5 mol L^−1^) NaOH solution and introduced into a 50 mL double-necked reaction flask, where one neck served for the injection of NaOH solution while the other facilitated the release of hydrogen gas. The reaction flask was immersed in a constant-temperature water bath maintained at 318 K to ensure temperature stability. The generated gas passed through a washing bottle to remove moisture before being weighed on an electronic balance connected to a computer for data recording. To minimize experimental error, each trial was performed under identical conditions and repeated at least twice; measurement errors were estimated to be less than 5%. Assuming complete recovery of all generated hydrogen, the hydrogen yield is defined as the ratio of the actual volume produced to the theoretical volume expected. The IHGR is defined as the volume of hydrogen produced per minute. This study presents and discusses the hydrolysis-based hydrogen generation process over a duration of 650 s.

## 4. Conclusions

In this study, DSSW-X composite powders were synthesized using a vibration grinding technique, The influences of the type of grinding agent, the amount of additive, and the duration of grinding on the hydrolysis of DSSW-X composite powder for hydrogen production and the kinetics were investigated. The results showed that the type of grinding agent, the amount of additive, and the duration of grinding had a great influence on the hydrolysis reaction. When the grinding agent was KCl, the grinding duration was 180 s, and the grinding agent content was 25 wt%, the hydrolysis of DSSW for hydrogen production performance was the best, the hydrogen yield reached 86.1%, and the IHGR was 399.37 mL min^−^^1^ (g DSSW)^−^^1^. At 338 K, the hydrolysis reaction achieved a hydrogen yield of 85% and an IHGR of 1383.6 mL min^−^^1^ (g DSSW)^−^^1^ within just 100 s. The increased addition of KCl significantly enhanced the reaction performance, which can be attributed to the dissolution of embedded KCl on the surface of DSSW in the solution, providing essential pores for the reaction. Concurrently, K^+^ formed a mixed alkali solution within NaOH, while Cl^−^ contributed to pitting corrosion during the reaction. Moreover, a grinding duration of 180 s yielded the smallest particle size, while a grinding agent content of 25% wt resulted in the highest surface roughness, which is conducive to the rapid hydrogen production process through hydrolysis. Kinetic studies indicate that during the rapid reaction phase, the chemical reaction rate serves as the rate-determining step, whereas in the slow reaction phase, the diffusion rate governs the movement of OH^−^ ions toward the surface of Si particles. The apparent activation energy is 45.62 kJ mol^−^^1^. The hydrolysis and hydrogen production process established by the shrinking nucleus model is:

$$1-{\left(1-x\right)}^{\frac{1}{3}}=5\times 10^{4}\times \exp\left(\frac{45.62}{\mathrm{RT}}\right)\times t$$

## Figures and Tables

**Table 1:** Fitted data for DSSW-KCl 25% wt samples at various temperatures based on the chemical control model and diffusion control model.

	First (k)	Stage (R^2^)	Second (k)	Stage (R^2^)
Temperature (K)	$1-{\left(1-x\right)}^{\frac{1}{3}}$			
308	8.53228 × 10^−4^	0.968	2.45195 × 10^−4^	0.974
318	0.00166	0.958	1.41306 × 10^−4^	0.906
328	0.00226	0.925	2.57262 × 10^−4^	0.954
338	0.00427	0.995	3.5813 × 10^−4^	0.961
Temperature (K)	$1-\frac{2}{3}x-{\left(1-x\right)}^{\frac{2}{3}}$			
308	2.09242 × 10^−4^	0.894	1.11003 × 10^−4^	0.978
318	4.52398 × 10^−4^	0.932	6.80189 × 10^−5^	0.910
328	7.9742 × 10^−4^	0.889	1.27913 × 10^−4^	0.956
338	0.00135	0.970	1.55666 × 10^−4^	0.950

## Data Availability

Data are contained within the article.

## References

[1] Mason J.E. (2007). World energy analysis: H_2_ now or later?. Energ. Policy.

[2] Soler L., Candela A.M., Macanás J., Muñoz M., Casado J. (2009). Hydrogen generation by aluminum corrosion in seawater promoted by suspensions of aluminum hydroxide. Int. J. Hydrogen Energy.

[3] Turner J., Sverdrup G., Mann M.K., Maness P.C., Kroposki B., Ghirardi M., Evans R.J., Blake D. (2008). Renewable hydrogen production. Int. J. Energ. Res..

[4] Fan M., Xu F., Sun L., Zhao J., Jiang T., Li W. (2008). Hydrolysis of ball milling Al–Bi–hydride and Al–Bi–salt mixture for hydrogen generation. J. Alloys Compd..

[5] Onwuemezie L., Darabkhani H.G. (2024). Biohydrogen production from solar and wind assisted af-mec coupled with mfc, pem electrolysis of H_2_O and H_2_ fuel cell for small-scale applications. Renew. Energ..

[6] Schlapbach L. (2002). Hydrogen as a fuel and its storage for mobility and transport. MRS Bull..

[7] Sakintuna B., Lamari-Darkrim F., Hirscher M. (2007). Metal hydride materials for solid hydrogen storage: A review. Int. J. Hydrogen Energy.

[8] Alinejad B., Mahmoodi K. (2009). A novel method for generating hydrogen by hydrolysis of highly activated aluminum nanoparticles in pure water. Int. J. Hydrogen Energy.

[9] Kravchenko O.V., Semenenko K.N., Bulychev B.M., Kalmykov K.B. (2005). Activation of aluminum metal and its reaction with water. J. Alloys Compd..

[10] Xu L., Ashraf S., Hu J., Edwards P.P., Jones M.O., Hadzifejzovic E., Foord J.S. (2016). Ball-milled si powder for the production of H_2_ from water for fuel cell applications. Int. J. Hydrogen Energy.

[11] Yoo H., Ryu H., Cho S., Han M., Bae K., Lee J. (2011). Effect of si content on H_2_ production using Al-Si alloy powders. Int. J. Hydrogen Energy.

[12] Erogbogbo F., Lin T., Tucciarone P.M., Lajoie K.M., Lai L., Patki G.D., Prasad P.N., Swihart M.T. (2013). On-demand hydrogen generation using nanosilicon: Splitting water without light, heat, or electricity. Nano Lett..

[13] Brack P., Dann S.E., Wijayantha K., Adcock P., Foster S. (2017). An assessment of the viability of hydrogen generation from the reaction of silicon powder and sodium hydroxide solution for portable applications. Int. J. Energ. Res..

[14] Kalamkar R., Yakkundi V., Gangal A. (2020). The consequence of silicon additive in isothermal decomposition of hydrides LiH, NaH, CaH_2_ and TiH_2_. Int. J. Hydrogen Energy.

[15] Brack P., Chillman M., Wijayantha K., Adcock P., Foster S., Dann S.E. (2017). Activation of silicon towards hydrogen generation by pelletisation. J. Alloys Compd..

[16] Gaffet E., Harmelin M. (1990). Crystal-amorphous phase transition induced by ball-milling in silicon. J. Less Common Met..

[17] Nilssen B.E., Kleiv R.A. (2020). Silicon powder properties produced in a planetary ball mill as a function of grinding time, grinding bead size and rotational speed. Silicon.

[18] Ding Z., Li Y., Yang H., Lu Y., Tan J., Li J., Li Q., Chen Y., Shaw L.L., Pan F. (2022). Tailoring MgH_2_ for hydrogen storage through nanoengineering and catalysis. J. Magnes. Alloys.

[19] Seidel H., Csepregi L., Heuberger A., Baumgärtel H. (1990). Anisotropic etching of crystalline silicon in alkaline solutions: I. Orientation dependence and behavior of passivation layers. J. Electrochem. Soc..

[20] Xia X., Ashruf C.M., French P.J., Rappich J., Kelly J.J. (2001). Etching and passivation of silicon in alkaline solution: A coupled chemical/electrochemical system. J. Phys. Chem. B.

[21] Liu Y., Wang X., Dong Z., Liu H., Li S., Ge H., Yan M. (2013). Hydrogen generation from the hydrolysis of mg powder ball-milled with AlCl_3_. Energy.

[22] Grosjean M., Zidoune M., Roué L., Huot J., Schulz R. (2004). Effect of ball milling on the corrosion resistance of magnesium in aqueous media. Electrochim. Acta.

[23] Imamura K., Kimura K., Fujie S., Kobayashi H. (2016). Hydrogen generation from water using si nanopowder fabricated from swarf. J. Nanopart. Res..

[24] Grosjean M., Zidoune M., Roué L., Huot J. (2006). Hydrogen production via hydrolysis reaction from ball-milled mg-based materials. Int. J. Hydrogen Energy.

[25] Mahmoodi K., Alinejad B. (2010). Enhancement of hydrogen generation rate in reaction of aluminum with water. Int. J. Hydrogen Energy.

[26] Hiraki T., Takeuchi M., Hisa M., Akiyama T. (2005). Hydrogen production from waste aluminum at different temperatures, with LCA. Mater. Trans..

[27] Fan M., Sun L., Xu F. (2010). Study of the controllable reactivity of aluminum alloys and their promising application for hydrogen generation. Energ. Convers. Manage..

[28] Yang H., Ding Z., Li Y., Li S., Wu P., Hou Q., Zheng Y., Gao B., Huo K., Du W. (2023). Recent advances in kinetic and thermodynamic regulation of magnesium hydride for hydrogen storage. Rare Met..

[29] Dry M.J., Bryson A.W. (1987). Kinetics of leaching of a low-grade FeNiCuCo matte in ferric sulphate solution. Hydrometallurgy.

[30] Song L., Di H., Yang K., Zhang L. (2022). Ultrasonic-enhanced sulfuric acid leaching kinetics of high-grade germanium-containing materials. Chem. Eng. Process. Process Intensif..

